# Synthesis and characterization of ZnO nanostructures using palm olein as biotemplate

**DOI:** 10.1186/1752-153X-7-71

**Published:** 2013-04-20

**Authors:** Donya Ramimoghadam, Mohd Zobir Bin Hussein, Yun Hin Taufiq-Yap

**Affiliations:** 1 Materials Synthesis and Characterization Laboratory (MSCL), Institute of Advanced Technology (ITMA), Universiti Putra Malaysia, 43400 UPM, Serdang, Selangor, Malaysia; 2 Research Center for Catalysis Science and Technology PutraCAT, Faculty of Science, Universiti Putra Malaysia, 43400 UPM, Serdang, Selangor, Malaysia

**Keywords:** Zinc oxide nanostructures, Palm olein, Biotemplate, Soft templating, Palm oil, Hydrothermal synthesis, ZnO

## Abstract

**Background:**

A green approach to synthesize nanomaterials using biotemplates has been subjected to intense research due to several advantages. Palm olein as a biotemplate offers the benefits of eco-friendliness, low-cost and scale-up for large scale production. Therefore, the effect of palm olein on morphology and surface properties of ZnO nanostructures were investigated.

**Results:**

The results indicate that palm olein as a biotemplate can be used to modify the shape and size of ZnO particles synthesized by hydrothermal method. Different morphology including flake-, flower- and three dimensional star-like structures were obtained. FTIR study indicated the reaction between carboxyl group of palm olein and zinc species had taken place. Specific surface area enhanced while no considerable change were observed in optical properties.

**Conclusion:**

*Phase-p*ure ZnO particles were successfully synthesized using palm olein as soft biotemplating agent by hydrothermal method. The physico-chemical properties of the resulting ZnO particles can be tuned using the ratio of palm olein to Zn cation.

## Background

Nanostructures and nanomaterials are subjected to a research focus in material science due to their unique properties and extensive applications. Among them, ZnO is of a great interest due to its wide band gap (3.37 eV) and large exciton binding energy (60 meV). It has wide applications in different industries including photodetectors [1], sensors [2], solar cells [3], antibacterial for medical products [4-6], cosmetics [7], etc. ZnO nanostructures can be synthesized by various chemical or physical methods such as precipitation [8], sol–gel [9], solvo/hydrothermal [10], chemical vapor deposition [11], spray pyrolysis [12], etc.

In fact, the specific properties of ZnO and its applications depend on its morphology, size and structure. Therefore, lately many researchers are more interested to explore the methods of synthesis of nanomaterials. To meet this requirement and along with developing the common synthesis methods of nanostructures, an increasing surge for utilizing bio-inspired synthesis, has been developed. A green approach for nanomaterials synthesis by applying biotemplate allows the reaction to proceed usually in milder conditions. In addition, due to their ability to form specific structures and self-assembling function, biomolecules may act as templates to synthesize functional nanomaterials of different morphologies. Based on this fact, lots of biomolecule-assisted syntheses of nanomaterials and nanocomposites have been reported recently. Some of them include L-cysteine, lysine [13-16], gelatin [17] and PEG [18], to name a few. Additionally, some biotemplates have been used for the synthesis of ZnO nanoparticles namely orange juice [19], albumen [20], cyclodextrin [21], egg-shell membrane [22], silk [23], peptide structures [24], DNA [25], pollen grain [26], wood [27], and different types of microorganisms [28-30]. In principle, the process of biotemplating can be described as seeking to either replicate the morphological characteristics and the functionality of a biological species or the use of biological structure to guide the assembly of inorganic materials [31], followed by removing of the template and finally forming a pure phase material with the required morphology.

A possible substitute for biotemplate material to mediate the morphologies of inorganic materials is palm olein. Palm olein is the liquid fraction of palm oil which is highly monosaturated and rich in oleic acids [32]. Generally, it contains 46% oleic acid, 37% palmitic acid, 11% linoleic acid, 4% stearic acid and 1% myristic acid. Two major grades of palm olein are produced in Malaysia, standard olein and double fraction (or super) olein [33]. Palm olein application extended from daily usage in the kitchen toward the industrial due to its excellent physical properties and oxidative stability. The use of palm olein to synthesize ZnO nanoparticles offers the benefits of eco-friendliness, low-cost and amenability for large scale production. Moreover, the existence of various functional groups such as hydroxyl and carboxylate should offer a wide variety of nucleation sites for surface controlled inorganic deposition, and could be used in the synthesis of inorganic materials.

Previous works showed palm oil and palm olein have been used extensively as source of carbon [34] and to produce biodiesel due to its rich sources of fatty acids [35]. There are several studies in which the effect of different fatty acids including oleic acid [36], ricinoleic acid [37] were investigated on the formation of nanostructure materials. It is assumed that the carboxyl head group of fatty acid will chemically bound to the surface of nanostructures and modify the surface structural property of particles [38]. To the best of our knowledge, the present study is the first report on ZnO nanoparticles synthesized using palm olein as biotemplate.

## Results and discussion

### XRD analysis

**Figure 1:**
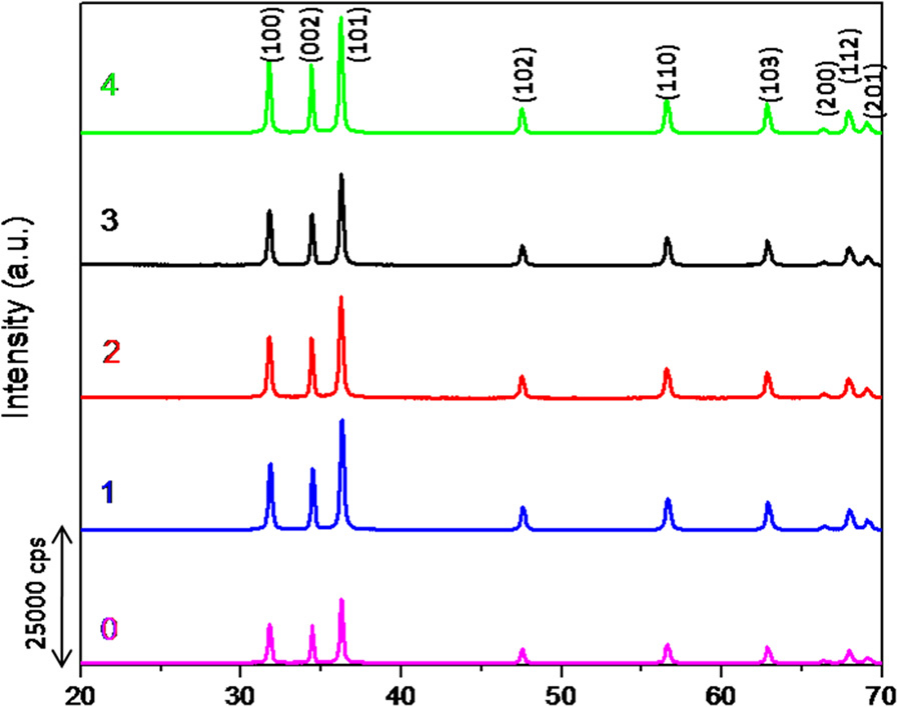
Powder X-ray diffraction (PXRD) patterns of as-prepared ZnO synthesized at different volumes (mL) of palm olein.

Figure 1 shows the XRD patterns of the samples synthesised at different volumes of palm olein (PO) by hydrothermal method. All the diffraction peaks can be indexed as hexagonal wurtzite-structure (JCPDS card No. 36–1451). The sharp and narrow peaks also illustrate that ZnO particles enjoy high crystallinity and purity.

In the case of ZnO samples synthesized using 8 and 16 mL of palm olein (PO) by CVD method, the pure phase of ZnO could not be achieved. Different peaks could be observed from the XRD patterns even after further treatment by two times calcinations at 500°C and 800°C for 5 and 3 hours, respectively. This shows that the pure phase of ZnO with high concentration of PO cannot be achieved through the CVD method under our experimental condidtion. Therefore we continue with the low concentration of PO through hydrothermal method.

### Morphology and size

**Figure 2:**
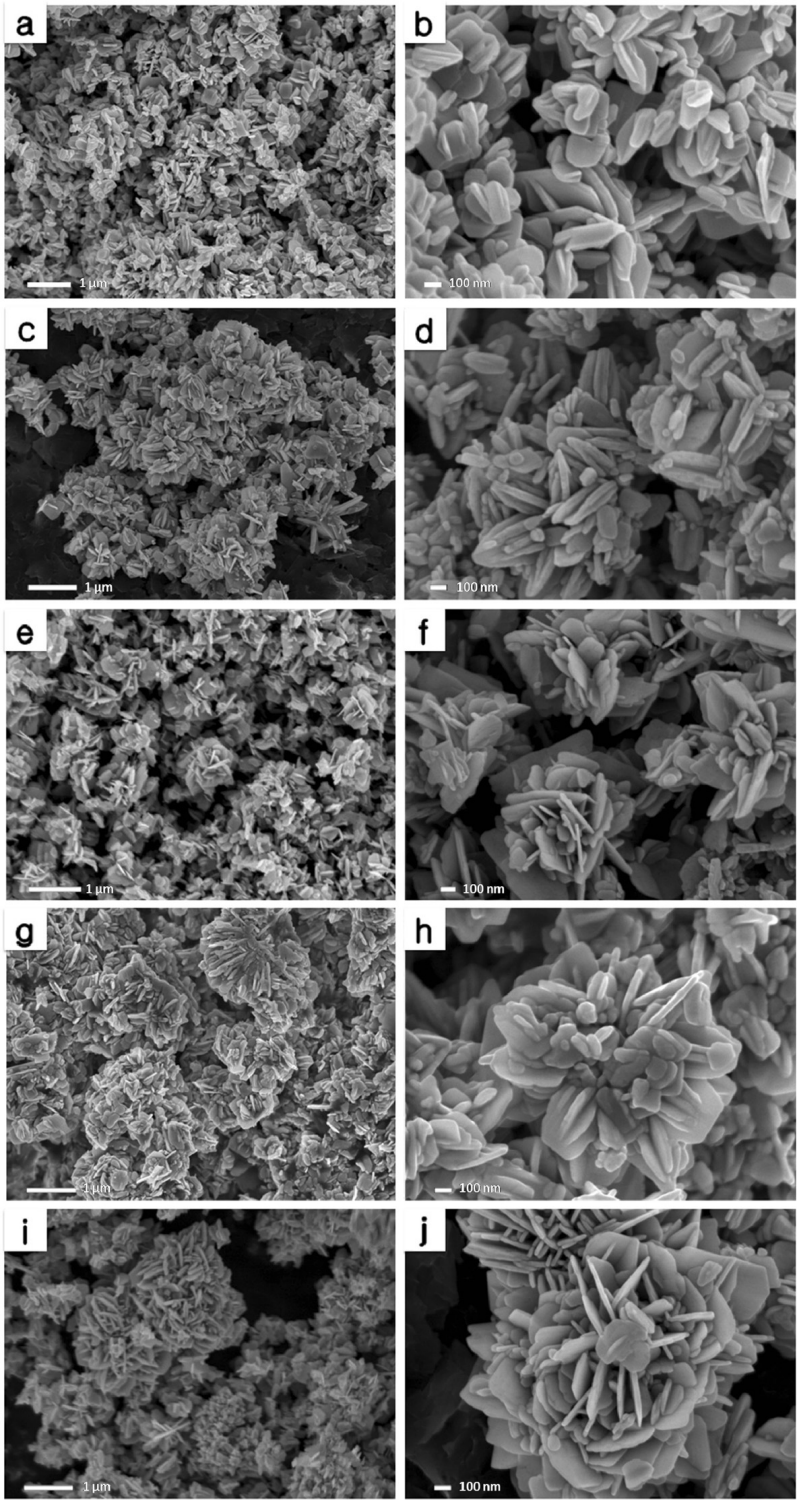
Field emission scanning electron microscopy (FESEM) images of ZnO prepared using different volumes of PO (mL); 0 (a, b), 1 (c, d), 2 (e, f), 3 (g, h), 4 (i, j).

Field emission scanning electron microscopy (FESEM) images of the as-synthesized ZnO nanostructures synthesized at different volumes of palm olein (PO) are shown in Figure 2. ZnO particles synthesized at 1 mL PO shown in Figure 2c and d are more ordered and regular-shaped compared with ZnO particles prepared without PO (a, b). As clearly seen from Figure 2d, the most of the particle’s shape consists of several plate-like sheets attaching together in the center in which ZnO crystal seed is located. It seems that, firstly the ZnO seeds formed by reaction between zinc hydroxide (Zn(OH)_2_) and fatty acids of palm olein (PO). The suggesting mechanism can be introduced in this way that fatty acids of palm olein assemble and form a spherical micelle around the zinc hydroxide crystals and lead to the formation of nucleation sites of ZnO. Therefore during the reaction time, with increasing the quantity of the growth unit of reactant, the ZnO nuclei will be subsequently increased in a way that conglomeration of the ZnO nuclei structure can lead to three-dimensional star-like particles. (Figure 2d).

Figure 2e and f images show ZnO sample synthesized at 2 mL PO. The morphology of the ZnO particles has been slightly changed in comparison with that of the ZnO synthesized using 1 mL PO, showing flower-like particles consisting of lots of nano- and micro-sized sheets. A closer look on the as-synthesized ZnO products using 1 and 2 mL PO, reveal that with increasing the concentration of PO, the size of particles increased but the diameter is dramatically decreased. The thin sheets of ZnO crystals assembled like petals of a flower from edge to centre.

FESEM images of ZnO particles synthesized using 3 mL PO are shown in Figure 2g and h. The overall shape of flower-like can be easily seen from the morphology of the particles. However, detailed view on the particles shows that similar structure of three-dimensional star-like particles appears in this sample. It is notable that particles seems to merge together and became embedded with the adjacent particles. Therefore the formation of the structure seems to be incomplete and even sometimes unclear. In addition, very much agglomerated structure can be seen from FESEM images in some areas. Another difference in the morphology of these products that can be taken into account is that corners of the sheets show curvature-like shape for ZnO particles prepared using 3 mL PO while those prepared using 2 mL PO shows that the corners of the sheets are clearly sharp and orthogonal.

ZnO particles prepared using 4 mL PO is shown in Figure 2i and j. Even though the structure of the particles demonstrates the same flower-like shape as seen in Figure 2j, the overall morphology has been changed to some extent. In other words, it contains lots of plate-like particles assembling laterally as petals of a flower while other particles located vertically in the centre.

Generally the mechanism of “biotemplate” can be described either by replicating the morphological characteristics and the functionality of a biological species or using a biological structure to guide the assembly of inorganic materials. In the case of palm olein, which is a complex biomacromolecular, biologically guided assembly of zinc nanostructures has been possibly occurred with the presence of PO functional groups. These functional groups are produced due to the hydrolysis of palm olein in water under the experimental condition stated in the experimental part, created nucleation sites for zinc crystal growth and then promote the pattern or certain morphologies formation. The self-assembly is guided by the presence of the biotemplate and is directed by covalent or non-covalent interactions or molecular recognition process [31]. The electrostatic interaction may occur between carboxyl and hydroxyl groups of PO and zinc cations. This electrostatic attraction may also occur between primary and secondary particles resulted in the formation of complex structures. It is noteworthy that changes in the concentration of PO in the mother liquor, resulted in changing the environment for the nucleation and reaction thermodynamics and subsequently leads to different assemblies for primary and tertiary particles and resulted in different morphologies.

Moreover, this template-assisted method is considered as soft-templating method since palm olein, an organic template, was used to synthesize mesoporous zinc oxide nanostructures. The formation of zinc oxide nanostructures can be summarized in three steps; first - the self-assembly of the palm olein, second - the organization of zinc acetate over the PO self-assembly in regular ordered array to form a stable inorganic-organic hybrid and finally, the successful removal of organic template to get a phase-pure zinc oxide.

**Figure 3:**
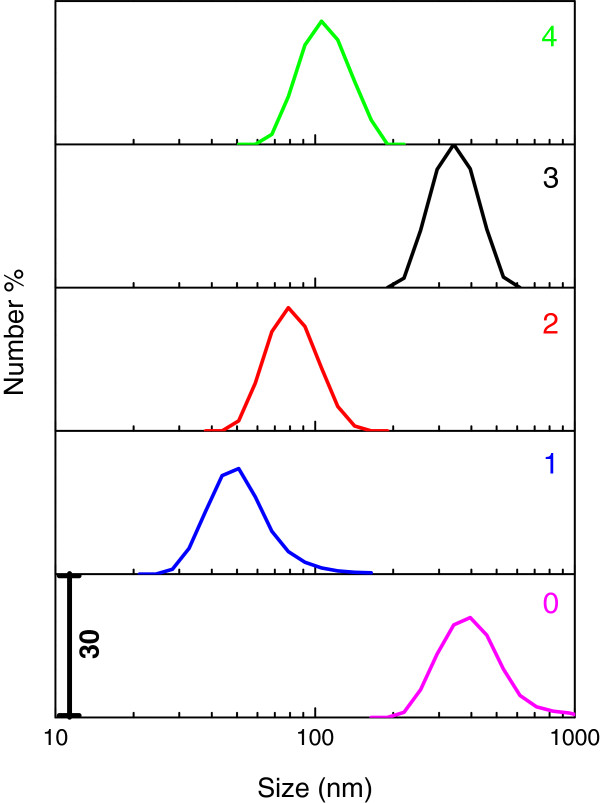
Particle size distribution of ZnO synthesized using different volumes of PO (0, 1, 2, 3, 4 mL).

Figure 3 shows the particle size distribution for ZnO synthesized using 1, 2, 3 and 4 mL palm olein. Particle size distribution of ZnO synthesized without palm olein is also represented as a reference. As seen in Figure 3, particle size for ZnO synthesized without PO shows distribution that at 200–1000 nm indicating micro-sized particles. However, the range has been dramatically decreased when the ZnO sample was synthesized using 1 mL PO. In other words, using 1 mL PO could change the size of ZnO from 20 nm to about 200 nm. Similarly, the particle size distribution for ZnO sample prepared at 2 mL PO demonstrates a slightly narrower distribution between 40–200 nm. Results from FESEM and particle size analysis reveal that using 2 mL palm olein, not only could change the morphology of the ZnO particles, but also reduced the size of the particles, considerably. In the case of ZnO sample synthesized at 3 mL PO, particle size has been sharply increased. On the basis of the results from particle size distribution, the range of 200–500 nm can be due to the agglomeration of particles which is in good agreement with FESEM results. Figure 3 clearly shows that the size of particles for ZnO sample prepared at 4 mL PO decreased considerably to as low as 50 nm, representing nano-sized particles.

Therefore, from particle size results show that ZnO samples synthesized with the presence of palm olein could lead to form both micro- and nanostructures. It is noteworthy that particles as small as 20 nm could be produced when palm olein was used as biotemplate.

### FTIR spectroscopy

To investigate the bio-template effect on the synthesis of ZnO nanoparticles prepared by hydrothermal method, FTIR spectra were measured at room temperature using the KBr pellet technique in the range of 4000–400 cm^−1^. Samples were gently mixed with 250 mg KBr powder and compressed into discs at a force of 13kN for 5 min using a manual tablet presser.

**Figure 4:**
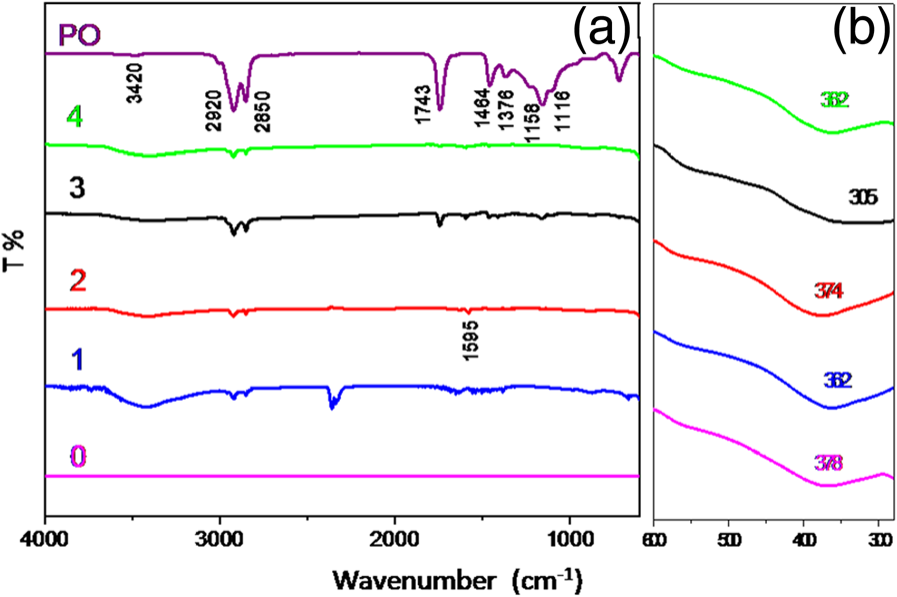
**FTIR spectra of ZnO samples synthesized at different volumes of PO (a) in the range of 4000–600 cm**^**-1 **^**and (b) in the range of 600–280 cm**^**-1**^**.**

**Figure 5:**
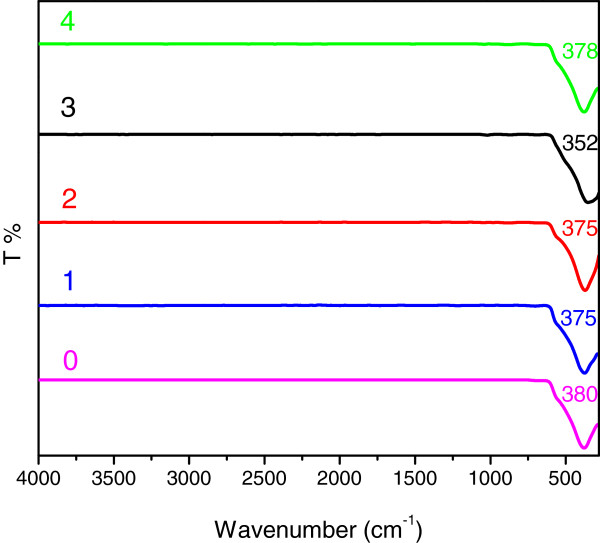
FTIR spectra of ZnO samples synthesized at different volumes of PO after calcinations at 500°C for 5 hours.

Figure 4 shows the FTIR spectra for the as-prepared ZnO nanoparticles using different volumes of PO together with palm olein as reference. As seen from Figure 4a, some peaks of palm olein can be clearly observed in FTIR spectra of the as-prepared ZnO nanoparticle, including absorption bands at 2850 and 2920 cm^-1^ which are assigned to asymmetric and symmetric stretching vibrations of CH_2_ group, respectively. Also bands at about 1743, 1464, 1376 and 1158 cm^-1^ which are attributed to C = O stretching, C-H scissoring, CH_3_ bending and C-O stretching, respectively, can be seen in FTIR spectra of as-synthesized ZnO nanoparticles. Bands at 3380–3420 cm^-1^ correspond to water, OH stretching vibration. In addition, a new band at about 1595 cm^-1^ is assigned to the stretching vibration of zinc carboxylate (COO-Zn), indicating successful reaction between the –COOH group of palm olein and the –OH group on the surface of ZnO nanoparticles [39]. In fact, Zn-OH group on the particle’s surface plays a role as a reaction site. However, from FTIR results for sample after calcinations at 500°C for 5 hours (Figure 5), indicate that the reaction between carboxylic and zinc hydroxide groups has been physically occurred at the particle surface, which cannot be removed during the washing process. The characteristic FTIR peaks, belong to biotemplate (PO) cannot be seen, which indicates that it was decomposed after calcinations treatment.

The characteristic peak of ZnO FTIR spectra are shown in Figure 4b which were recorded separately in the range of 600–280 cm^-1^. The peaks at about 300–370 cm^-1^ are in good agreement with the observation of previous work [40]. As seen from Figure 5, ZnO characteristic peaks have shifted to higher wavenumbers from 350–380 cm^-1^ which is much closer to the absorption peak of commercial ZnO (380 cm^-1^).

### Thermal analysis

**Figure 6:**
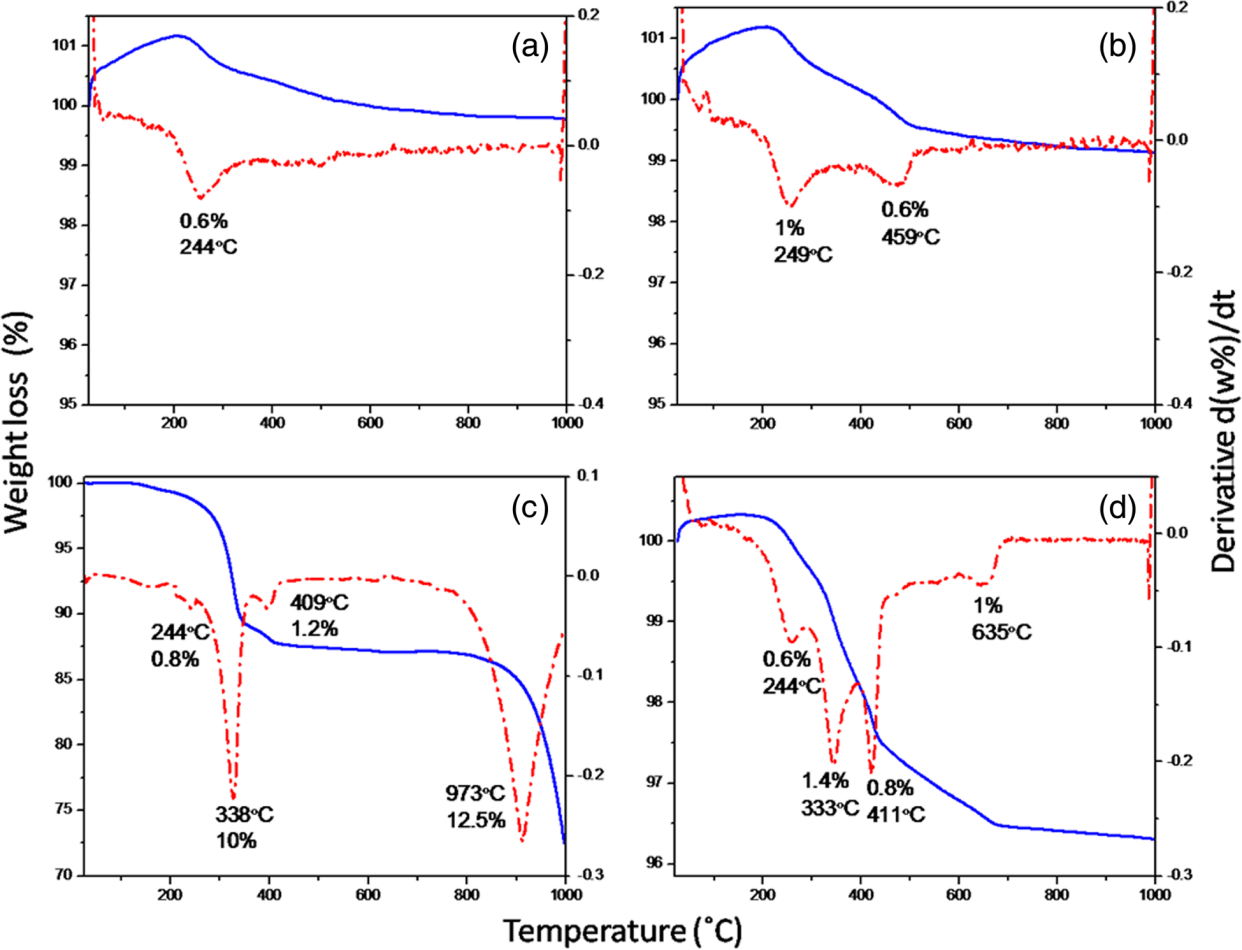
Thermogravimetric and differential thermogravimetric thermogram (TGA-DTG) of as-synthesized ZnO samples prepared at different volumes of (a) 1, (b) 2, (c) 3 and (d) 4 mL PO.

Thermal analysis of the as-synthesized ZnO samples synthesized using different volumes of palm olein (1, 2, 3 and 4 mL) is shown in Figure 6. As seen in the Figure 6, the first weight loss step with maximum peak at about 244°C corresponds to thermal decomposition of zinc carboxylate compound. This peak can be clearly observed in all the thermograms. Weight loss percentages for samples synthesized using 1, 2, 3 and 4 mL PO is 0.6, 1, 0.8 and 0.6%, respectively. This indicate that small amount of zinc carboxylate has been produced during the reaction.

The second weight loss, which can be clearly observed for sample synthesized using 3 and 4 mL PO at about 335°C is assigned to fatty acids decomposition. The percentage of weight loss is 10.0 and 1.4% for sample synthesized at 3 and 4 mL PO, respectively. In the case of samples prepared using 1 and 2 mL PO, peaks are not observed probably due to the presence of a little amount of PO which is still remain in the sample.

The third weight loss occurs at about 410°C is attributed to mono-/diglyceride [41]. This step includes 0.6, 1.2 and 0.8% weight loss percentages for samples synthesized at 2, 3 and 4 mL PO, respectively. As seen from Figure 6a, DTG curve shows very weak peak at this temperature for the sample synthesized at 1 mL PO.

Finally, the last weight loss of 1% at 634°C is due to dehydroxylation of residual palm olein. This step has been shifted to higher temperature, 970°C, for the sample synthesized at 3 mL PO which can be due to the high heating rate. However, it can be easily interpreted from the curve that the degradation has not been leveled off possibly even after 1000°C. Therefore, the high percentage of 25% weight loss demonstrates that sample prepared at 3 mL PO adsorbed the highest amount of palm olein which is in good agreement with the FTIR results. It should be noted that high heating rate of 10°/min is resulted in overlapping, shifting or even disappearing of the peaks as mentioned earlier.

### Surface properties

**Figure 7:**
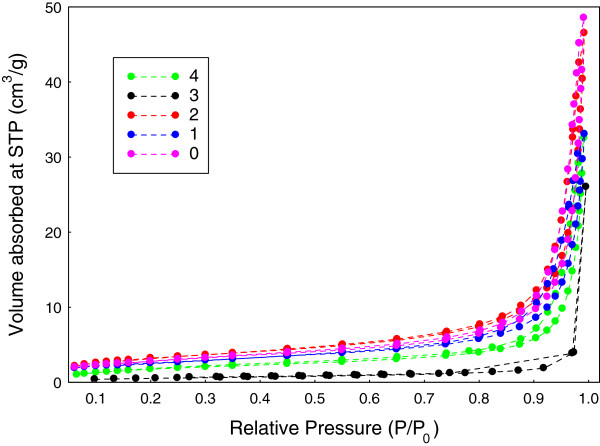
Nitrogen adsorption-desorption isotherms of as-obtained ZnO nanostructures synthesized at different volumes of PO.

The adsorption-desorption isotherms for ZnO nanoparticles prepared at different volumes of palm olein are shown in Figure 7. All the isotherms can be ascribed as Type IV according to IUPAC classification. Moreover, their hysteresis loops are of Type H3, indicating mesoporous materials. On the basis of the results shown in Figure 7, the absorption of ZnO sample synthesized using 1 mL PO showing a gradual increased in the volume adsorbed from low relative pressure of about 0.06 to 0.6 and then followed by a sharp rise from 0.6 and above. However, the values of volume absorbed for sample synthesized using 1 mL PO, are lower than those of ZnO sample prepared without PO. On the other hand, the opposite results can be clearly observed for ZnO sample synthesized using 2 mL PO. The adsorption which starts from 0.06 is higher compared to the ZnO synthesized without PO until near to relative pressure of 0.98. Similarly, ZnO sample prepared using 4 mL PO shows similar behavior to the sample synthesized at 1 mL PO. As shown in the Figure 7, the as-synthesized sample prepared using 3 mL PO shows that the absorption started to increase from a relative pressure of about 0.1 and then increased slowly, until the relative pressure of about 0.9, followed by a sudden increased from 0.9 to 0.99. This sample has the lowest volume absorbed among all the samples. The most volume absorbed can be observed for the sample prepared at 0 mL of PO followed by 2 mL PO.

The desorption branch of isotherms are quite similar, due to similarity in their pore’s texture, though the ZnO sample synthesized at 3 mL PO shows a different desorption branch. The adsorption-desorption isotherm of sample synthesized using 3 mL PO indicates that the evaporation of the condensed liquid from pores is delayed. Therefore, the access of adjacent pores to the vapor phase can be achieved instead [42]. As a result, the desorption branch does not follow the original hysteresis loop.

**Figure 8:**
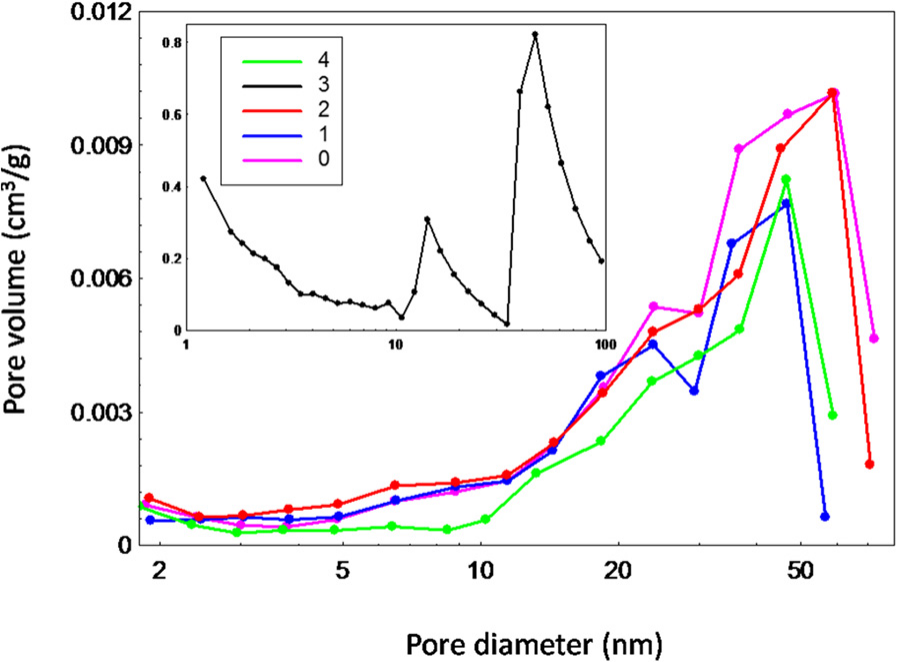
Barret-Joyner-Halenda (BJH) pore size distribution of as-synthesized ZnO nanostructures synthesized using different volumes of PO.

Figure 8 shows the Barret-Joyner-Halenda (BJH) pore size distribution for the as-synthesized ZnO nanostructures using different volumes of palm olein. As clearly seen from the plots, pores are located mainly between 2–50 nm revealing that the material is dominated by mesoporous structure, which is in good agreement with the adsorption isotherm of Type IV. Pore size distribution of as-synthesized ZnO particles without PO is also given for comparison, showing rather similar characteristics to those of ZnO synthesized with the present of PO, especially the one using 1 mL PO. However, it is not uniform, and this suggests a dual mesoporous distribution around two sizes of 25 and 60 nm. The pore sizes of ZnO samples prepared using 1, 2 and 4 mL PO are distributed at around 48, 60 and 48 nm, respectively.

More uniform pores can be observed for the ZnO samples prepared using PO compared to the one synthesized without PO. The ZnO samples synthesized using 1 and 2 mL PO show higher pore volume in smaller pore range (2–20 nm) and lower pore volumes with bigger pore range (20–70 nm), respectively compared to the sample prepared without PO. The pore size distribution for sample synthesized at 3 mL PO is shown in Figure 8 (inset). A dual mesoporous distribution at 14 and 46 nm can be clearly seen. The overall shape of the size distribution for the sample prepared using 3 mL PO is different from the others indicating different pore texture. Moreover, in the case of ZnO synthesized using 4 mL PO, a narrowest pore size distribution was observed which demonstrates the formation of more uniform pores.

**Figure 9:**
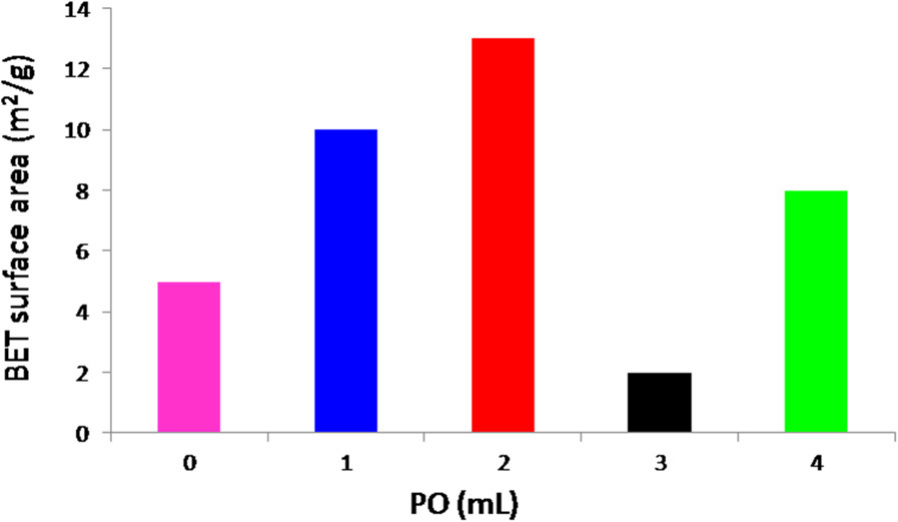
BET surface area values of ZnO nanostructures synthesized at different volumes of PO (0, 1, 2, 3 and 4 mL).

The BET surface area values of ZnO synthesized at different volumes of PO are illustrated in Figure 9. Based on surface area values, it can be derived that PO can be used as biotemplate to increase the surface area of the ZnO nanostructures. As shown in Figure 9, BET surface area of 10 and 13 m^2^/g was obtained for the sample prepared using 1 and 2 mL PO, respectively, an increase of two and three folds, respectively compared to the value of 5 m^2^/g for the ZnO synthesized without any biotemplate. In the case of sample synthesized using 3mL PO, the decrease of surface area was observed, possibly due to the collapse of pore structure which occurred as a result of merging and embedding the particles with adjacent particles as mentioned earlier (see Thermal analysis) [32].

### Optical properties

**Figure 10:**
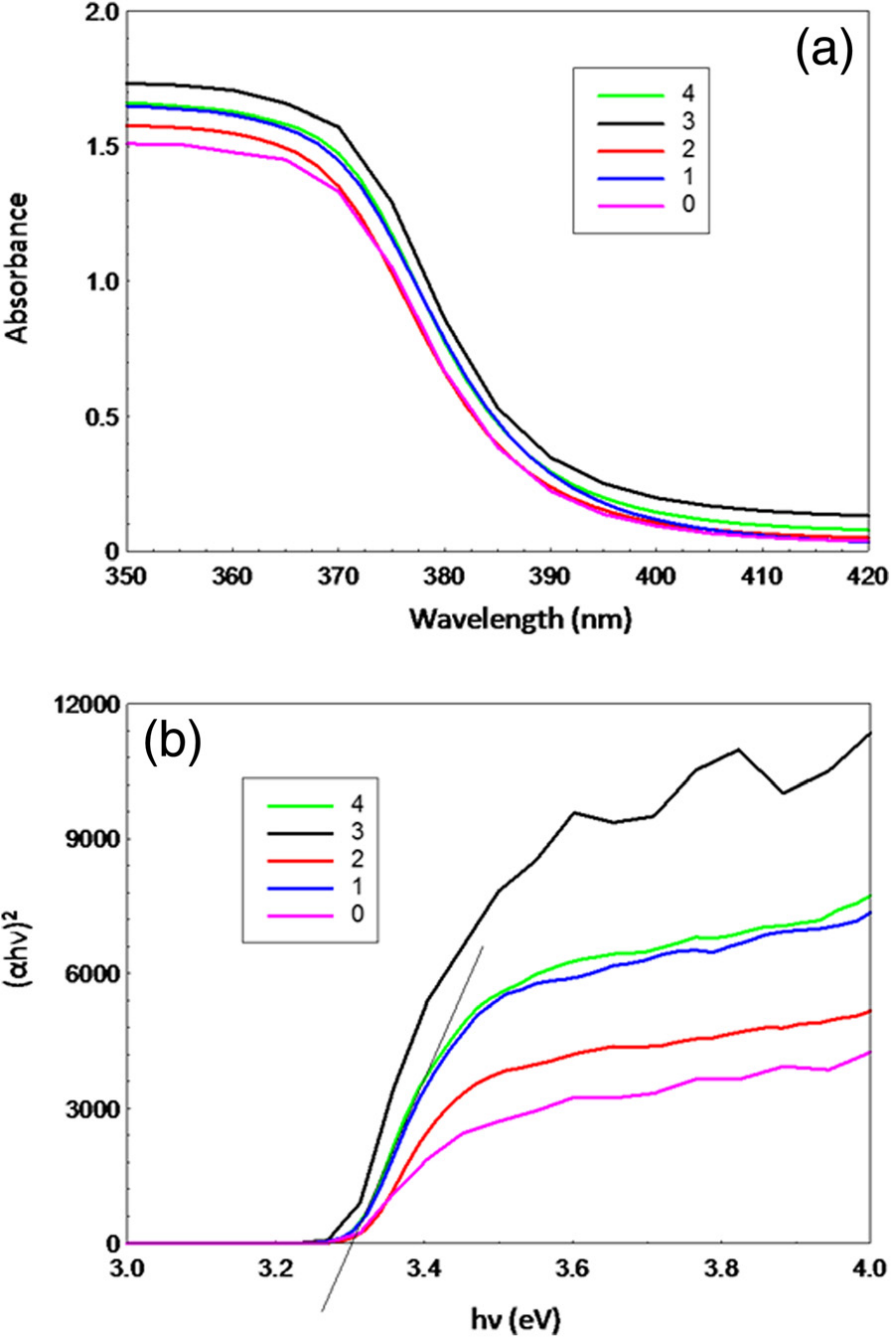
UV-vis absorption spectra (a) and Band gap energy (b) of as-synthesized ZnO nanostructures synthesized at different volumes of PO.

The UV–vis absorption spectra of as-synthesized ZnO nanostructures are shown in Figure 10a. All the curves show absorptions below 400 nm corresponding to the intrinsic band gap of ZnO which is related to electron transitions from the valence band to conduction band. In addition, the samples synthesized at different volumes of PO indicate higher UV–vis absorption compared to the one synthesized without PO. The direct-band gap energies as shown in Figure 10b were estimated from the plots of the transformed Kubelka-Munk function, (αhυ)^2^ versus the photon energy (hυ). As shown in Figure 10b, linear region of the plot can be extrapolated to intersect the x-axis, and this value is identified as E_g_, the band gap energy. The E_g_ of the ZnO samples, syntheisised using 0, 1, 2, 3 and 4 ml PO were found to be very similar, 3.29, 3.31, 3.32, 3.30 and 3.3, respectively.

### Experimental procedure

All chemicals used in this work were of analytical reagent grade and used as received without any further purification. All the aqueous solutions were prepared using deionized water. Palm Olein (PO) was purchased from a market in Malaysia.

In a typical procedure, 1 g of zinc acetate (Zn(Ac)_2_.2H_2_O) and 0.8 g sodium hydroxide (NaOH) were dissolved in 25 mL distilled water under constant stirring (Zn^2+^ : OH^-^ = 1: 4). The measured pH was 13. After 1 hour stirring, different volumes of palm olein, 0, 1, 2, 3, 4, 8 and 16 mL were introduced to the solution (i.e. the ratio of zinc acetate to palm olein was 1:0, 1:1, 1:2, 1:3, 1:4, 1:8 and 1:16, w/v%) and stirring was continued until palm olein was completely dissolved, i.e. a flocculent precipitate rather white in color was obtained. Finally, the mentioned solution was transferred into a Teflon-lined stainless steel autoclave, 50 mL and hydrothermal growth was carried out at 120°C for 18 h. After treatment, the autoclaves were allowed to cool down and the precipitates were collected, centrifuged and the supernatant was discarded. The obtained particles were washed three times with ethanol and distilled water, in order to remove impurities and dried at 60°C for 24 h.

Samples with high concentrations of palm olein (8 and 16 ml PO) were not able to dry at 60°C even after several days. The solutions were oily and sticky. Therefore we changed the hydrothermal method to chemical vapor deposition (CVD). After preparing the solution as discussed earlier, the yellowish solution was transferred into ceramic boat. Then the boat was placed at the center of the furnace’s tube. The process of heating was performed for 2 hours at 500°C under N_2_ gas atmosphere. Then samples were allowed to cool down and the precipitates were collected and ground to powder form.

### Characterization

Powder X-ray diffraction (PXRD) analysis was performed on a Shimadzu diffractometer, XRD-6000 (Tokyo, Japan) equipped with CuK_α_ radiation. The morphology of the micro- and nanostructures were characterized by a field emission scanning electron microscopy (FESEM) a JOEL JSM-6400 (Tokyo, Japan). Particles Size distribution was analyzed by a Malvern zetasizer nano series ZEN1600 (Worcestershire, UK). Fourier transform infrared spectra were recorded over the 280–4000 cm^−1^ range using a Perkin-Elmer 100 spectrophotometer (Waltham, MA, USA) under standard conditions. Thermogravimetric and differential thermogravimetric analyzer were carried out using a Mettler Toledo instrument (Greifensee, Switzerland) using heating rate of 10°C/min, in the range of 25–1000°C under nitrogen atmosphere. Surface characterization of the material was carried out using nitrogen gas adsorption-desorption technique at 77 K by a Micromeritics ASAP 2000 (Norcross, GA, USA). The UV-VIS-NIR spectrophotometer UV-3600 SHIMADZU was used to determine the optical properties.

## Conclusion

Pure phase ZnO particles were successfully synthesized using palm olein as soft biotemplating agent, which lead to form both micro- and nano-structure particles. Different morphologies, namely flower-, flake- and star-like particles could be obtained. The morphology changes can be possibly due to the reaction between carboxylic group of palm olein and zinc hydroxide groups, which has been physically occurred on the particle surface. Moreover, maximum weight loss of approximately 25% was observed due to the template degradation. In addition, biotemplate could be also used to modify the surface properties of ZnO particles.

## Competing interest

There is no conflict of interest for all authors of this article.

## Authors’ contributions

DR is the first author of this article. MZBH is the second and correspond author. YHTY is the third author. All authors read and approved the final manuscript.
